# In-Situ Coating of Iron with a Conducting Polymer, Polypyrrole, as a Promise for Corrosion Protection

**DOI:** 10.3390/ma17194783

**Published:** 2024-09-29

**Authors:** Jaroslav Stejskal, Marek Jurča, Miroslava Trchová, Jan Prokeš, Ivo Křivka

**Affiliations:** 1 University Institute, Tomas Bata University in Zlin, 760 01 Zlin, Czech Republic; jurca@utb.cz; 2 Central Laboratories, University of Chemistry and Technology, Prague, 166 28 Prague 6, Czech Republic; miroslava.trchova@vscht.cz; 3 Charles University, Faculty of Mathematics and Physics, 180 00 Prague 8, Czech Republic; jprokes@semi.mff.cuni.cz (J.P.); krivka@semi.mff.cuni.cz (I.K.)

**Keywords:** iron, carbonyl iron microparticles, polypyrrole, conducting polymer, hybrid core–shell composites, resistivity, conductivity, magnetic properties, corrosion protection

## Abstract

Iron microparticles were coated with polypyrrole in situ during the chemical oxidation of pyrrole with ammonium peroxydisulfate in aqueous medium. A series of hybrid organic/inorganic core–shell materials were prepared with 30–76 wt% iron content. Polypyrrole coating was revealed by scanning electron microscopy, and its molecular structure and completeness were proved by FTIR and Raman spectroscopies. The composites of polypyrrole/carbonyl iron were obtained as powders and characterized with respect to their electrical properties. Their resistivity was monitored by the four-point van der Pauw method under 0.01–10 MPa pressure. In an apparent paradox, the resistivity of composites increased from the units Ω cm for neat polypyrrole to thousands Ω cm for the highest iron content despite the high conductivity of iron. This means that composite conductivity is controlled by the electrical properties of the polypyrrole matrix. The change of sample size during the compression was also recorded and provides a parameter reflecting the mechanical properties of composites. In addition to conductivity, the composites displayed magnetic properties afforded by the presence of iron. The study also illustrates the feasibility of the polypyrrole coating on macroscopic objects, demonstrated by an iron nail, and offers potential application in the corrosion protection of iron. The differences in the morphology of micro- and macroscopic polypyrrole objects are described.

## 1. Introduction

Hybrid organic/inorganic composites based on a core–shell combination of conducting and magnetic components introduce a class of functional materials with a wide application potential. The organic part may be represented by conducting polymers and the inorganic moiety by metals or ferrites. The typical preparation strategy is illustrated by the coating of metal microparticles with a conducting polymer, here of carbonyl iron with polypyrrole.

Carbonyl iron, a highly pure iron, is industrially produced by the thermal decomposition of liquid iron pentacarbonyl, Fe(CO_5_). It is typically obtained and distributed as a powder composed of spherical microparticles. Most authors felt the need to protect iron surfaces from corrosion before the deposition of polypyrrole. The iron surface was coated with an overlayer to protect the metal from corrosion, e.g., by silanization [1,2]. Alternatively, carbonyl iron was treated with hydrochloric acid to introduce hydroxyl groups onto the surface [3,4] that act as potential grafting sites for polypyrrole, or the iron surface was etched with dilute nitric acid [5]. The grafting of carbonyl iron with poly(pyrrole-functionalized ethyl methacrylate) also falls into this category [6]. The surface of iron microparticles was coated with carbon nanotubes using polypyrrole as a binder [7]. Carbonyl iron was also modified with a protective layer of polydopamine [8] or silica [2,9] before polypyrrole deposition.

The direct coating with polypyrrole has also been reported [10,11,12]. The in-situ deposition of polypyrrole onto the substrates immersed in aqueous reaction mixture takes place during the oxidation of pyrrole to polypyrrole. Any such interfaces become coated with a polypyrrole overlayer without any pretreatment of substrates. The interaction between polypyrrole and the coated substrate is based on physical adsorption of oligomeric pyrrole intermediates followed by the growth of polypyrrole chains. The feasibility of such approach is illustrated in the present study for the coating of iron microspheres.

Hybrid polypyrrole/carbonyl iron composites have found an application mainly in two fields. The first concerns the electromagnetic interference shielding in the GHz microwave region where the functional components are required to be conductive and/or magnetic [13,14,15]. Iron provides magnetic properties and its conductivity is supplemented by the incorporation of polypyrrole. The low density of conducting polymers compared with metals is favourable for the design of lightweight shielding materials, e.g., aerogels [8], often required by the aerospace sector. The composites usually include an additional component, such as multi-wall carbon nanotubes [7], graphene oxide [15], or nanocarbon [16]. These are incorporated as fillers in a carrier polymer matrix that provides the desired mechanical and utility properties.

Magnetorheology is the second field of potential application. The fluids used in magnetorheology are based on the suspension of magnetic particles in a liquid medium. They increase their viscosity in response to the applied magnetic field. In addition to magnetic moieties, conducting polymers often play a part in their design [17]. Carbonyl iron microspheres coated with polypyrrole represent a simple system to investigate [4,6,11]. Polypyrrole coating decreases the average particle density and prevents the dispersed phase from sedimentation. A mixture of carbonyl iron microparticles with polypyrrole nanotubes decorated with magnetite nanoparticles constitutes a bidisperse magnetorheological fluid [18]. The sophisticated combination of small and large ferromagnetic objects improves the rheology response under applied magnetic field.

Corrosion protection of metals is a prospective field of research [19]. The corrosion of metals is a redox process associated with the transfer of electrons from metal to oxidant. Conducting polymers are electroactive, i.e., they are able to be oxidized and reduced. They may participate in parallel redox processes, and thus affect or inhibit the corrosion [20,21,22,23,24]. Conducting polymers, such as polyaniline or polypyrrole, are polycations balanced with counter-anions [25], which can also be active in associated ionic processes.

The role of the conductivity of conducting polymers in corrosion phenomena, however, is not obvious. Electronic and proton conductivity is specific for this class of polymers [26]. Under standard conditions, the molecules of oxidant and reductant have to meet in order to undergo redox reaction. If, however, both moieties are in contact at different spots on a conducting polymer, they can react with each other by transferring electrons and protons through the polymer body, without the need for oxidant and reductant molecules to physically meet [27,28]. This explains why associated redox processes can easily proceed in solid state [29,30] or in frozen aqueous media [31,32], where the diffusion of reactant molecules is restricted.

One comment is pertinent. Iron, indeed, suffers from corrosion, which affects its composite properties in an undesirable manner in general and its long-term stability in particular. There are two facets of the problem to consider. The application of conducting polymers, such as polypyrrole, in the corrosion protection of metals is a positive message [33,34]. On the other hand, the preparation of polypyrrole is typically achieved by the oxidation of pyrrole with iron(III) chloride [7,8,14,15,18], i.e., under acidic conditions. In this case, the partial or complete dissolution of iron takes place. Even if some iron were left, chloride counter-ions in polypyrrole would promote the future corrosion of iron. The coating of iron microspheres was also simply achieved by dispersion in dilute nitric acid [5]. A partial dissolution of iron took place generating iron(III) cations that acted as an oxidant of pyrrole. In order to reduce the corrosion by acids, the oxidation of pyrrole with ammonium peroxydisulfate [4,6,11,35] is a preferred preparation route.

The present study does not concern directly the corrosion protection of metals; there are no corrosion experiments performed or discussed. On the contrary, it is aimed at the design of new materials based on the chemical coating of iron surfaces at the micro- and macroscale, and the characterization of resulting composites with respect to their electrical and magnetic properties. Such materials, however, would find applications in corrosion protection when chemical deposition is preferred over the currently used electrochemical process. A knowledge of electrical properties is essential for the understanding of protection performance.

Conducting polymers are not just conducting [36]. They are also electroactive and applicable in energy storage [37,38], and may act as efficient adsorbents of pollutant dyes [39] or flame retardants [40]. Hybrid composites including a magnetic component find uses in corrosion protection coatings [41], heating elements with enhanced thermal conductivity and heat capacity, or in the biosciences [42], viz. in tissue regeneration [43]. Electrical conductivity, however, always remains a key parameter of composites. It has rarely been reported for powdered materials in the literature due to the experimental difficulties met in its determination, because often the composites cannot be compressed to the free-standing objects required by routine methods. A new experimental way to determine resistivity as a function of applied pressure is offered and the results discussed in the present study.

## 2. Experimental

### 2.1. Preparation

Carbonyl iron microspheres (SQ type) of average diameter D50 4–5 μm with a broad size distribution and purity 99.5 wt% were supplied by BASF (Ludwigshafen, Germany). Various amounts of iron (2–8 g) were suspended in 100 mL water containing pyrrole. Ammonium peroxydisulfate solution of the same volume was added under stirring conditions at room temperature. The 200 mL of reaction mixture contained 0.1 M pyrrole (1.34 g, 20 mmol) and 0.125 M ammonium peroxydisulfate (5.71 g, 25 mmol). Monomer and oxidant were of reagent grade purchased from Sigma Aldrich branch (Prague, Czech Republic). The polymerization of pyrrole gave 1.40 g of polypyrrole sulfate; the stoichiometric expectation was 1.78 g for the completely protonated form [44]. After 10 min, the microparticles coated with polypyrrole were separated by filtration and copiously rinsed with water followed by ethanol to remove any soluble species. The solids were left to dry at ambient temperature in open air for 48 h. The same protocol was used for the deposition of polypyrrole on the iron nail.

### 2.2. Characterization

A scanning electron microscope (Tescan Vega, Brno, Czech Republic) was used to display the morphology. The composition of composites with polypyrrole was determined after the combustion of the organic part in oxygen atmosphere at 800 °C in a muffle furnace (Nabertherm L9/S27, Lilienthal, Germany). The content of iron was calculated from the mass of the residual solids composed of iron oxides [45].

ATR FTIR spectra were analysed using a Nicolet 6700 spectrometer (Thermo-Nicolet, Waltham, MA, USA) in the 4000–400 cm^−1^ range at the resolution 4 cm^−1^, 64 scans, and Happ-Genzel apodization. Raman spectra were registered with a Thermo Scientific DXR Raman microscope (Thermo Fisher Scientific, Waltham, MA, USA) with a 780 nm laser line. The scattered light was analysed by a spectrograph with a holographic grating of 400 lines per mm, a pinhole width of 50 μm, and an acquisition time of 10 s with 10 repetitions. Magnetic hysteresis curves were recorded with a vibrating sample magnetometer (VSM, Model 7407, Westerville, OH, USA).

The DC resistivity of composites was determined by a four-point van der Pauw method using a lab-made press operating with a cylindrical glass cell 10 mm in diameter. A current source Keithley 220, a Keithley 2010 multimeter, and a Keithley 705 scanner with a Keithley 7052 matrix card were included in the setup. Powders were compressed with a glass piston carrying four platinum/rhodium electrodes at the perimeter, and resistivity was recorded as a function of applied pressure. The pressure up to 10 MPa (=102 kp cm^−2^) was applied with an E87H4-B05 stepper motor (Haydon Switch & Instrument Inc., Waterbury, CT, USA) and registered with a L6E3 strain gauge cell (Zemic Europe BV, Etten-Leur, The Netherlands). The sample thickness was monitored during the compression with a dial indicator Mitutoyo ID-S112X (Mitutoyo Corp., Sakado, Japan).

## 3. Results and Discussion

### 3.1. Composites

**Figure 1:**
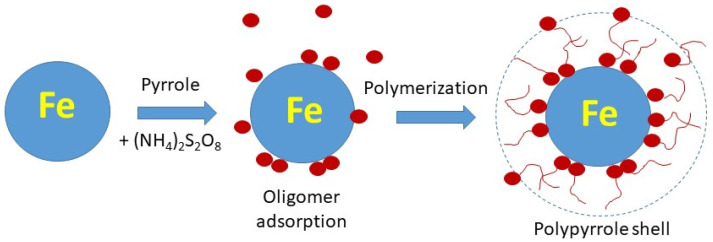
Pyrrole oligomers produced by the oxidation of pyrrole adsorb on the surface of iron core and subsequently start the brush-like growth of polypyrrole chains that produce the shell.

Various substrates are easily modified at the surface by the overlayer of a conducting polymer, such as polyaniline or polypyrrole [46]. This happens when these polymers are prepared by the oxidation of respective monomers in acidic aqueous medium. During this process, the hydrophobic oligomers produced in the early stages of oxidation adsorb at any available interface immersed in the reaction mixture (Figure 1). They subsequently start the brush-like growth of polymer chains that results in the coating, e.g., here of iron microspheres. The typical thickness of the coating is 100–200 nm and can be varied by the concentration of reactants, acidity of medium, some additives, and temperature [46].

The typical oxidation of pyrrole to polypyrrole takes place in water in the presence of strong oxidants. It is therefore essential that the substrate to be coated is stable under such conditions and does not extensively corrode or dissolve. Iron(III) chloride is a typical oxidant used for the preparation of polypyrrole [14,15,18] and provides the conducting polymer with a conductivity of the order of units S cm^−1^. If iron is present in the reaction mixture under such conditions, it readily dissolves. The metal dissolution subsequently increases in pH, and the polymerization of pyrrole that requires acidic medium becomes complicated and may yield pyrrole oligomers instead of polypyrrole. The situation, however, becomes more favourable when ammonium peroxydisulfate is used and only limited dissolution of metal is found. It seems that at first iron dissolves in acidic medium, but this process stops once polypyrrole deposits on its surface. This oxidant has been used therefore for the surface deposition of polypyrrole on iron microparticles (Table 1).

The preparation of pyrrole used in the present study expected a stoichiometric yield of 1.78 g of polypyrrole sulfate [44]; in practice, 1.40 g of polypyrrole was obtained due to the uncertainty in the degree of polymer protonation and potential formation of soluble by-products. The composite yield increased after iron was added to the reaction mixture, but only to ≈60% of the expectation (1.40 g polypyrrole + added g of iron). This means that part of the iron still dissolved during polypyrrole deposition, in contrast to, e.g., nickel [44]. The content of iron varied from 30 to 76 wt% (Table 1). Due to a large difference in the densities of components, the volume fractions of iron are considerably lower.

### 3.2. Morphology

**Figure 2:**
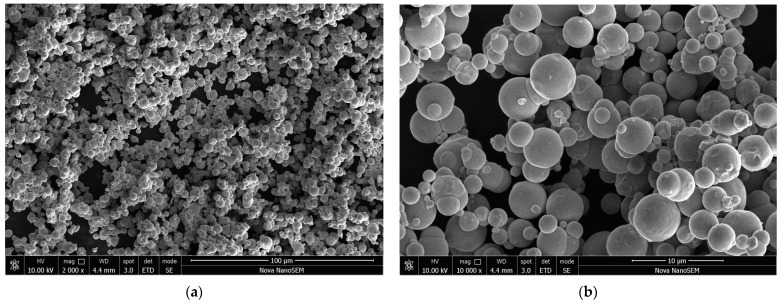
Carbonyl iron microspheres. Two magnifications: scale bars 100 μm (**a**) and 10 μm (**b**).

**Figure 3:**
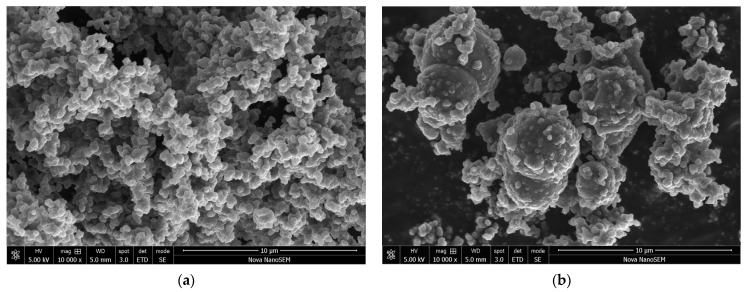
(**a**) Globular polypyrrole **coating** and (**b**) iron microparticles coated with polypyrrole (30 wt% iron).

**Figure 4:**
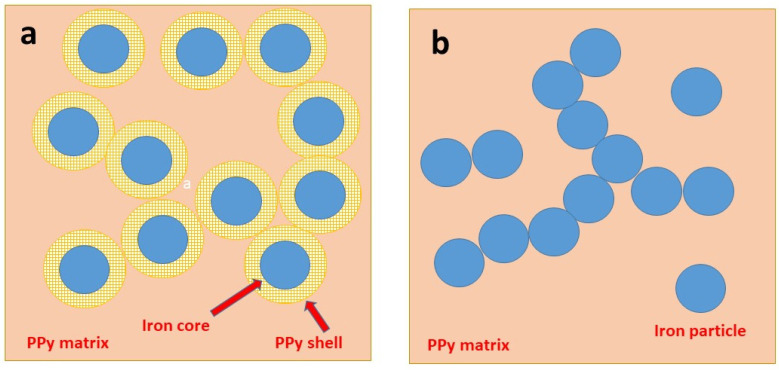
(**a**) Polypyrrole-coated iron microparticles embedded in a matrix of accompanying polypyrrole compared with (**b**) a simple mixture of iron microspheres with polypyrrole.

Carbonyl iron was supplied as microspheres with a diameter of several micrometres and a broad distribution of particle sizes (Figure 2). After the deposition of polypyrrole, in most places only its globular form, nanoparticles at a size in the order of 100 nm were observed (Figure 3a). The volume fraction of the conducting polymer considerably exceeded the fraction of iron in all samples (Table 1). Iron microparticles were thus completely embedded in the polypyrrole matrix (Figure 4). Individual iron microparticles with deposited polypyrrole were also found in the samples (Figure 3b).

### 3.3. FTIR Spectra

**Figure 5:**
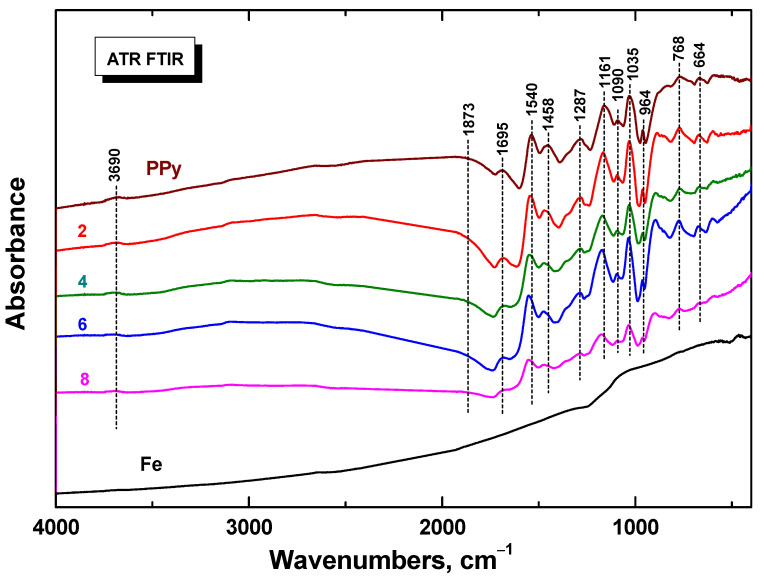
ATR FTIR spectra of polypyrrole/iron composites in dependence on iron mass entering the preparation (in g/200 mL). Cf. Table 1 for corresponding weight fraction of iron, *w*, in the composite. The spectra of the individual components are included for comparison.

Infrared spectra of composites in dependence on iron content obtained in ATR reflection mode (Figure 5) corresponded to the spectrum of the initial polypyrrole powder (spectrum PPy). The maxima of the main bands were well detected at 1695, 1540, 1458, 1287, 1161, 1090, 1035, 964, 768, and 664 cm^−1^ [44]. The spectrum of iron is featureless and does not exhibit an absorption peak. The shape of spectra slightly change with an increasing amount of iron in the reaction mixture. The intensity of the broad absorption band at wavenumbers above 2000 cm^−1^ (polaron band) decreased in correlation with the decreasing conductivity of the composites. This is also supported by a slight shift of the bands at 1540, 1458, and 1161 cm^−1^ to the higher wavenumbers, which is characteristic for the deprotonation of polypyrrole [47].

### 3.4. Raman Spectra

**Figure 6:**
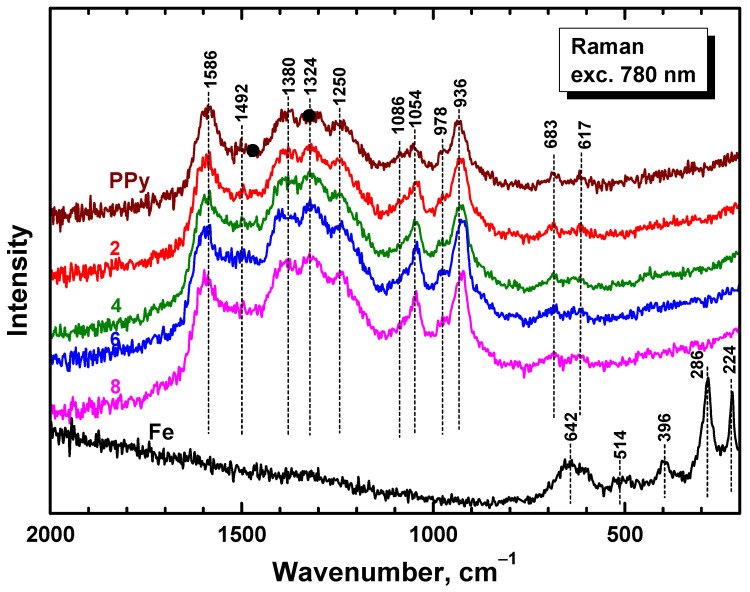
Raman spectra of PPy/Fe composites in dependence on iron mass entering the preparation (in g/200 mL).

Raman spectra of PPy/Fe composites in dependence on iron mass entering the preparation (Figure 6) exhibited only the main bands of neat PPy (spectrum PPy) situated at 1586, 1492, 1380, 1324, 1250, 1086, 1054, 978, 936, 683, and 611 cm^−1^ [47]. The laser excitation wavelength 780 nm was in resonance with the energy of delocalized polarons and bipolarons of polypyrrole [47]. The penetration depth was reduced to a few tens of nanometers and the Raman scattering occurred only at the surface of the polymer. The spectra are relatively noisy due to the low laser power used not to burn the thin films deposited on iron microparticles. No relatively sharp Raman peaks of iron (spectrum Fe) were detected in the spectra of composites, and this supports the concept of the completeness of polypyrrole coating (Figure 1).

### 3.5. Electrical Properties

**Figure 7:**
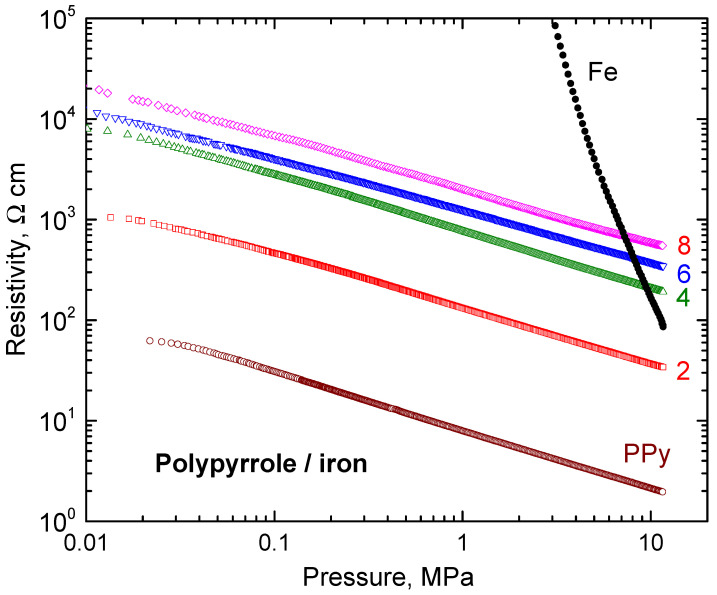
The pressure dependences of resistivity of composites in dependence on iron mass entering the preparation (in g per 200 mL). Cf. Table 1 for corresponding fractions of iron in composites. The plots of individual components are included for comparison.

The conductivity of bulk iron is 9.94 × 10^4^ S cm^−1^. The apparent conductivity of neat iron powder compressed at 10 MPa is about seven orders of magnitude lower, 5.78 × 10^−3^ S cm^−1^ (Table 2), due to the limited contact area of microspheres and interfacial barriers. Although materials scientists prefer to express electrical properties in terms of conductivity, physicists use the presentation of its reciprocal value, the resistivity (Figure 7). The latter parameter for carbonyl iron steeply decreased with applied pressure but did not reach the resistivity of bulk metal.

A similar trend is observed for all composites (Figure 7). The pressure dependences, *p*, of resistivity, *ρ*, are close to linear in double-logarithmic presentation, log *ρ* = A + B log *p*, A and B being parameters. They are less steep compared to iron (Table 2), and electrical properties are clearly controlled by the polypyrrole matrix (Figure 4). The parameter *B* provides the information about how fast the powders decrease their resistivity during compression and can be regarded as a measure of composite fluffiness.

If the composite powders can be compressed to free-standing pellets (here at 527 MPa), the conductivity is routinely determined by the four-point method (Table 2). Such conductivity values are about one order of magnitude higher compared to those determined under 10 MPa pressure.

Iron is more conducting than polypyrrole. There is an apparent paradox: when more conducting iron particles were introduced to the composite, the resistivity (and not the conductivity) increased (Figure 7). This is explained as follows: the iron particles are prevented from mutual contact by the polypyrrole coating and cannot create conducting pathways even at high loading (Figure 4a) in the contrast to simple mixtures (Figure 4b). The conduction is thus provided by the polypyrrole matrix. With the increasing volume fraction of iron, the fraction of polypyrrole decreased and, consequently, the resistivity increased as observed (Figure 7). An analogous trend has recently been reported for nickel microparticles coated with polypyrrole [44].

### 3.6. Mechanical Properties

**Figure 8:**
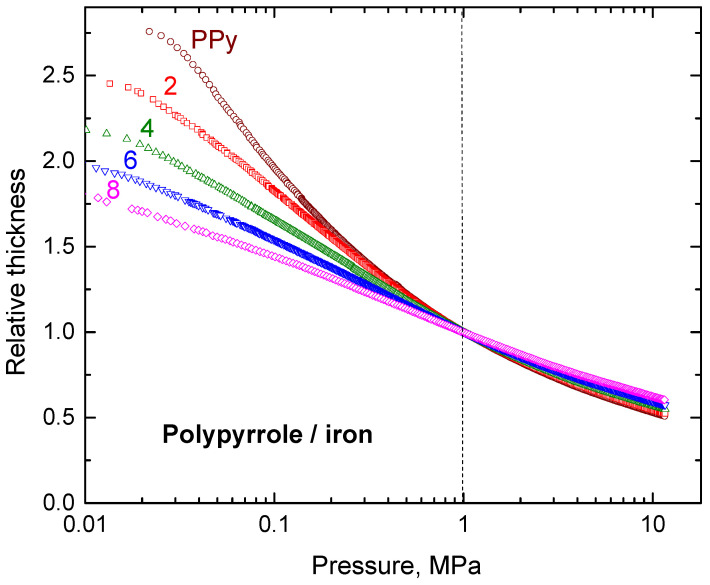
Pressure dependences of sample thickness during the compression relative to 1 MPa for various iron mass entering the preparation (in 0–8 g/200 mL).

The present experimental determination of resistivity also allows for the assessment of the mechanical properties of composite powders (Figure 8). Polypyrrole alone has a fluffy consistency. It is easily compressed, and the sample thickness is readily reduced at low pressures. With the increasing content of iron, the composites become stiffer, and the change of the thickness at increasing pressure is less pronounced. The behaviour of all samples is similar at pressures above 1 MPa.

### 3.7. Magnetic Properties

**Figure 9:**
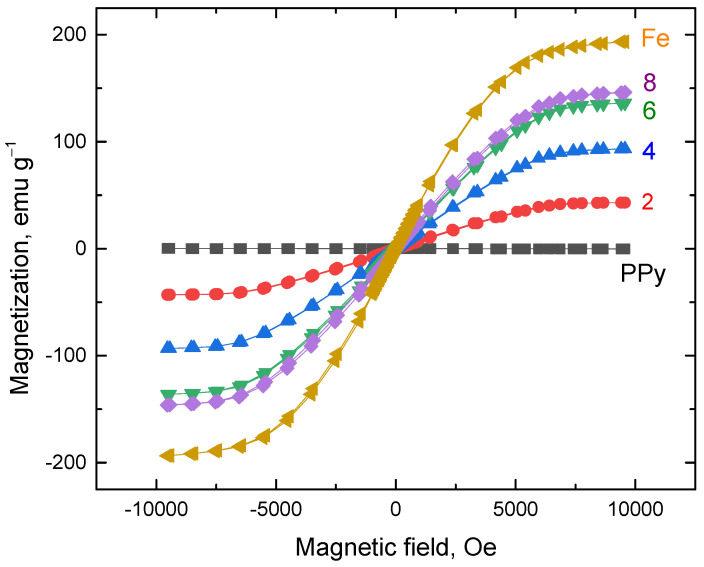
Magnetization curves of composites for various contents of iron in the reaction mixture (in g/200 mL).

As anticipated, the highest magnetization value of 193 emu g^−1^ (Table 2, Figure 9) was observed in the neat carbonyl iron. Given that polypyrrole does not exhibit intrinsic magnetic properties, the saturation and remanence magnetization of the composite materials are primarily determined by the iron content, in accordance with the hysteresis curves. For example, the composite prepared with 4 g of iron contains about ≈60 wt% of this metal (Table 1) and achieves about half the remanence and magnetic saturation compared to the carbonyl iron powder. The coercivity and remanence values (Table 2), along with the shape of the magnetization curve, indicate that the composites behave as magnetically soft materials, like the carbonyl iron itself. No significant differences in coercivity have been detected.

A comment is relevant: in addition to the coating of iron microparticles with polypyrrole, free polypyrrole generated outside them will be a part of the resulting composite powder (Figure 4a). Macroscopically, however, the composite behaves as homogeneous, i.e., it is attracted to the permanent magnet as a whole and it does not seem to contain any separate free non-magnetic polypyrrole part. We speculate that the polypyrrole chains produced outside iron particles are intertwined with those constituting the coating.

### 3.8. Coating of Macroscopic Iron Object

The above results demonstrate the feasibility of coating iron microspheres with a conducting polymer. This technique can be extended to macroscopic objects. In addition to conductivity, polypyrrole is also electroactive, i.e., it can be oxidized or reduced [19]. It is therefore able to participate in various redox reactions, the metal corrosion being an example, and its corrosion protection worth is to be considered.

**Figure 10:**
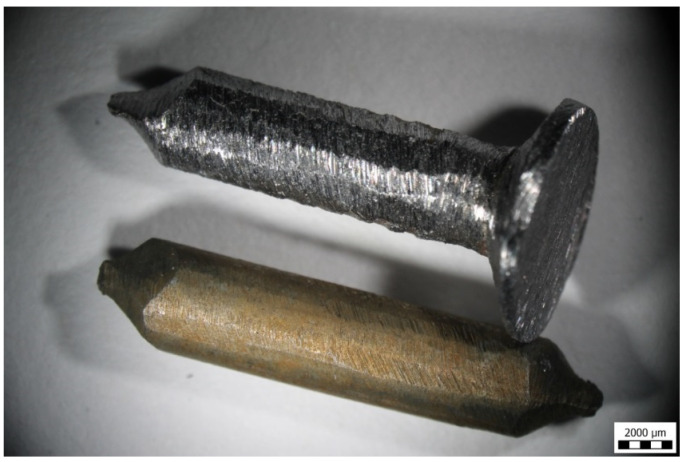
Parts of the iron nail before (**top**) and after coating with polypyrrole (**bottom**).

There is limited literature on polypyrrole corrosion protective coatings of iron surfaces, but its anticorrosion performance has been reported. In this case, however, polypyrrole was deposited on iron electrochemically [41,48,49,50,51,52,53]. The similar protection of aluminium has also been recently described [54]. The present study demonstrates the feasibility of an alternative way of polypyrrole coating of macroscopic iron objects based on chemical deposition. If, instead of iron microspheres, an iron nail was immersed in the reaction mixture, its surface became coated with polypyrrole (Figure 10). The deposited polypyrrole had good adhesion after drying.

**Figure 11:**
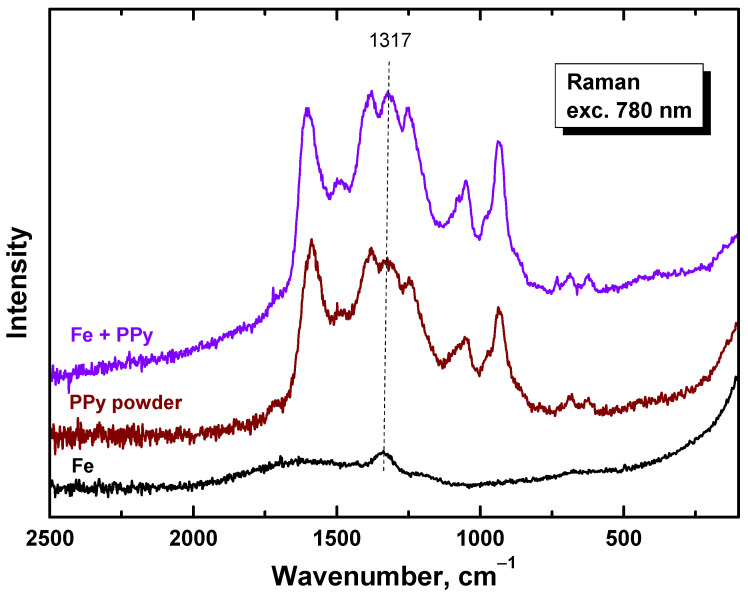
Raman spectra of the iron nail coated with polypyrrole, free polypyrrole powder, and original nail.

Raman spectra prove that the iron surface was coated with polypyrrole, and the molecular structure of the coating does not differ from free polypyrrole powder (Figure 11). The presence of pyrrole oligomers is suspected to accompany polypyrrole (see below), but they can hardly be distinguished from the polymer by this method. No new peaks in the spectra of the coating were present. This also indicated the absence of corrosion products which would manifest themselves by bands of iron oxides. The absence of the peak of iron at 1317 cm^−1^ in the spectrum of the coated nail could be regarded a proof of the coating completeness, but unfortunately it interferes with the band of polypyrrole in the same position.

**Figure 12:**
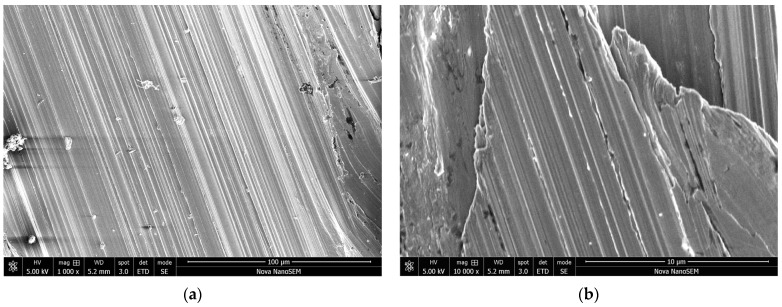
The surface morphology of the iron nail at two magnifications. Scale bars 100 μm (**a**) and 10 μm (**b**).

**Figure 13:**
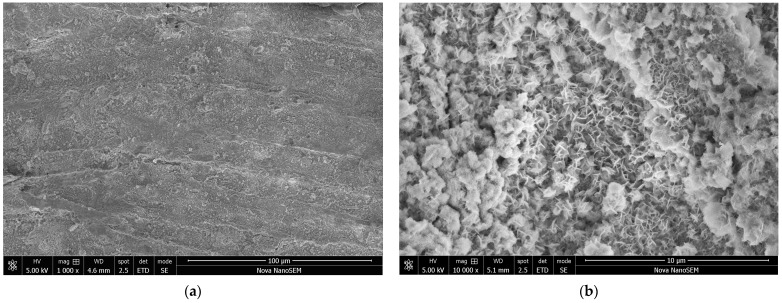
The surface morphology of the polypyrrole-coated iron nail at two magnifications. Scale bars 100 μm (**a**) and 10 μm (**b**).

Electron microscopy provides better insights into the coating morphology. The original surface of the nail (Figure 12) becomes clearly coated with polypyrrole (Figure 13a), but the detailed morphology is not uniform (Figure 13b) and it differs from the coating of carbonyl iron microspheres (Figure 3b). Whereas Figure 3b showed carbonyl iron particles coated with relatively regular globular polypyrrole particles, the coating of the nail creates significantly different and more complex morphology (Figure 13b). It is composed of irregular globular particles with radial needle-like protrusions growing out of their surface and interconnecting them.

**Figure 14:**
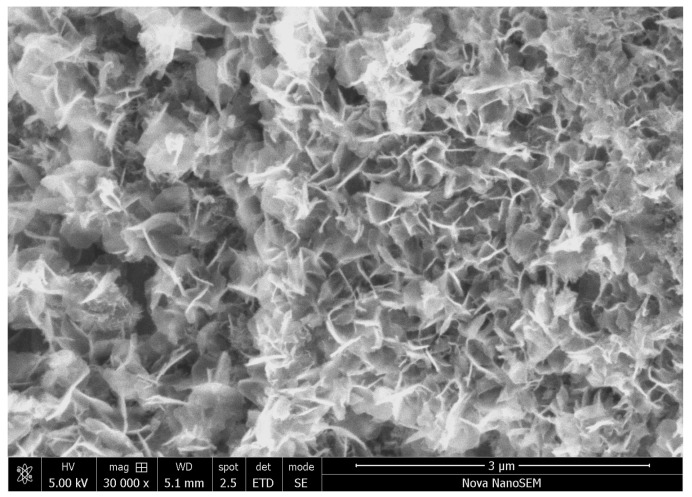
The network of oligomeric pyrrole nanoplates.

In addition to globular polypyrrole, there are regions composed of thin nanoplates organized in a sponge-like network (Figure 14). Similar and often spectacular morphologies have been reported for oligomers associated with other conducting polymers, viz. polyaniline [55,56,57]. They are probably non-conducting, but still electroactive, and may participate in the electrochemistry of corrosion processes, similar to corresponding polymers [58,59,60,61,62].

## 4. Conclusions

The present study illustrates the feasibility of the chemical coating of iron microspheres with a conducting polymer, polypyrrole, which is an alternative to electrochemical deposition. This was achieved on immersed substrates during the chemical oxidation of pyrrole with ammonium peroxydisulfate in aqueous medium. The resulting composite materials, polypyrrole-coated iron microspheres, were obtained as powders and characterized with respect to their electrical and magnetic properties. The conductivity of individual composites decreased (i.e., resistivity increased), despite the increasing content of iron. Since iron is a good metallic conductor, this is an apparent paradox. As iron microparticles are coated with polypyrrole, they cannot produce the metallic pathways and thus do not contribute to the overall conductivity of a composite. Polypyrrole coating is expected to protect the iron core from corrosion when the composites are used in electromagnetic interference shielding compositions or other applications. It is further demonstrated that a macroscopic object, such as an iron nail, can be similarly coated with polypyrrole. This observation opens the prospects to corrosion protection of iron surfaces coated with this conducting polymer.

## Figures and Tables

**Table 1:** Composite yield obtained and compared to the per cent of expectation, weight fraction of iron in the composite, *w*, and corresponding volume fraction, *φ*, depending on mass of carbonyl iron entering the preparation, *Fe* (in g per 200 mL).

*Fe*	Yield, g	Yield, %	*w*, wt% Fe	*φ*, vol% Fe
0 (PPy)	1.40	100	0	0
2	2.03	53.7	30.4	7.7
4	3.29	60.8	56.7	20.0
6	4.62	62.3	70.6	31.5
8	5.93	63.1	76.0	37.7

**Table 2:** Conductivity of free-standing composite pellets, σ*, and at 10 MPa pressure, *σ*_10_, the slope of the pressure dependence of resistivity, *B*, and magnetic properties of composites depending on mass of iron in the reaction mixture, *Fe* (in g per 200 mL).

*Fe*	σ*, S cm^−1^	*σ*_10_, S cm^−1^	−*B*	Coercitivity, Oe	Remanence, emu g^−1^	Saturation Magnetization, emu g^−1^
0	1.35	0.470	0.580	–	–	–
2	0.120	2.72 × 10^−2^	0.550	6.72	0.061	43.1
4	2.67 × 10^−2^	4.83 × 10^−3^	0.566	6.02	0.102	93.3
6	1.77 × 10^−2^	2.67 × 10^−3^	0.514	5.94	0.147	136
8	9.63 × 10^−3^	1.70 × 10^−3^	0.536	5.87	0.160	146
Fe	–	5.78 × 10^−3^	4.89	4.92	0.216	193

## Data Availability

The original contributions presented in the study are included in the article, further inquiries can be directed to the corresponding author.
